# Early Recurrence of HCC Is Driven by Inflammation-Related HIF-1α Independent Angiogenesis Rather than Hypoxia-Induced Immune Escape

**DOI:** 10.3390/biom16050723

**Published:** 2026-05-14

**Authors:** Lianda Siregar, Rino Alvani Gani, Toar J. M. Lalisang, Irsan Hasan, Suhendro, Heriawan Soejono, Siti Boedina Kresno, Nurjati Chairani Siregar, Muhammad Begawan Bestari

**Affiliations:** 1 Department of Internal Medicine, Faculty of Medicine, Universitas Indonesia, Jakarta 10430, Indonesia; rino.gani@ui.ac.id (R.A.G.); irsan.hasan@ui.ac.id (I.H.); suhendro@ui.ac.id (S.); czeresna.heriawan@ui.ac.id (H.S.); 2 Department of Digestive Surgery, Faculty of Medicine, Universitas Indonesia, Jakarta 10430, Indonesia; toar.m@ui.ac.id; 3 Department of Clinical Pathology, Faculty of Medicine, Universitas Indonesia, Jakarta 10430, Indonesia; sbkresno@gmail.com; 4 Department of Pathological Anatomy, Faculty of Medicine, Universitas Indonesia, Jakarta 10430, Indonesia; nurjati.chairani@ui.ac.id; 5 Department of Internal Medicine, Faculty of Medicine, Universitas Padjajaran, Bandung 40161, Indonesia; begawan@unpad.ac.id

**Keywords:** angiogenesis, CD4, CD8, early recurrence, hepatocellular carcinoma, hypoxia, PD-1, PDL1, T regulation, VEGF

## Abstract

Background: Hepatocellular carcinoma (HCC) shows a high rate of early recurrence after curative resection, indicating a critical contribution of tumor microenvironment-driven molecular mechanisms. Early recurrence of hepatocellular carcinoma is defined as recurrence within 6 months after curative resection, with a prevalence exceeding 30%. Hypoxia signaling and immune dysregulation have been implicated, yet their compartment-specific relevance remains unclear. Methods: This multicenter nested case–control study included 49 HCC patients to evaluate associations between hypoxia-inducible factor-1 alpha (HIF-1α), vascular endothelial growth factor (VEGF), tumor-infiltrating lymphocytes (TILs), CD4^+^ T cells, CD8^+^ T cells, regulatory T cells (Tregs), programmed cell death protein 1 (PD-1), and programmed death-ligand 1 (PD-L1) and early recurrence after resection. TIL density was assessed using hematoxylin and eosin staining, while immunohistochemistry was performed to quantify intratumoral and peritumoral expression of the studied markers. Receiver operating characteristic (ROC) curve analysis was used to evaluate the predictive performance. Recurrence-free survival (RFS) was analyzed using the Kaplan–Meier, and independent predictors were identified using multivariate Cox proportional hazards regression. Results: Early recurrence occurred in 11 of 49 patients (22.4%) of Child–Pugh A patients. Recurrent tumors were characterized by elevated VEGF expression despite absent HIF-1α, alongside significant depletion of intratumoral TILs (HR 5.02; 95% CI 1.09–23.26), CD4^+^ (HR 7.68; 95% CI 1.66–35.60) and CD8^+^ cells (HR 6.68; 95% CI 1.77–25.23) and reduced peritumoral CD8^+^ infiltration (HR 4.20; 95% CI 1.11–15.91). Multivariable analysis identified low intratumoral CD4^+^ (HR 7.98; 95% CI 1.63–39.07) and reduced peritumoral CD8^+^ expression (HR 4.98; 95% CI 1.14–21.70) as independent predictors, whereas HIF-1α, VEGF, Treg, PD-1, and PD-L1 were not significantly associated. Conclusions: Early HCC recurrence shows HIF-1α-independent angiogenesis alongside spatial immune depletion, supporting integrated immune profiling over single angiogenic markers.

## 1. Introduction

Hepatocellular carcinoma (HCC) is characterized by a high rate of early post-resection recurrence, which remains a major clinical challenge even when surgical resection is performed at an early stage. Early recurrence is commonly defined as tumor relapse occurring within ≤2 years after curative therapy and is generally associated with poorer prognosis. In contrast, late recurrence (>2 years) is more often considered to represent de novo (multicentric) tumor development arising independently in a carcinogenic liver background, such as cirrhosis, chronic inflammation, or metabolic liver disease [1,2].

However, the conventional 2-year threshold is increasingly considered too broad to meaningfully reflect the clinical and biological behavior of tumor recurrence. Several studies have suggested that shorter timeframes, such as ≤6–12 months, provide superior prognostic discrimination between biologically aggressive tumors and more indolent recurrence patterns [3,4,5]. Recurrence risk has been shown to peak at approximately 6 months, with a secondary peak around 24 months [6].

Consistently, previous studies have reported varying but convergent early recurrence patterns. A 1-year cut-off has been associated with up to 55.1% of recurrence cases [5]. Another study reported an 8-month threshold as the peak period of early HCC recurrence, affecting 35.5% of patients [4]. In addition, more recent findings indicate that over 30% of patients experience recurrence within the first 6 months after resection [3]. Overall, post-recurrence survival analyses have identified 6 months as an optimal cut-off point for distinguishing early recurrence associated with more aggressive tumor biology following resection [3]. Furthermore, post-recurrence survival analyses have reinforced 6 months as an optimal threshold for defining highly aggressive early recurrence. From a biological perspective, very early recurrence following resection, particularly within ≤6 months, is more frequently associated with pre-existing intrahepatic micrometastases that were present prior to surgery but remained undetectable by radiological assessment [7].

Tumor hypoxia is a hallmark of solid malignancies and triggers the activation of hypoxia-inducible factor-1α (HIF-1α), a key transcriptional regulator that induces the expression of multiple pro-angiogenic genes, including vascular endothelial growth factor (VEGF). Accumulating evidence indicates that HIF-1α contributes to hepatocellular carcinoma progression by promoting tumor invasiveness, cancer cell migration, angiogenesis, and overall tumor aggressiveness through the regulation of downstream target genes and associated molecular pathways [8].

HIF-1α-induced VEGF expression represents a canonical angiogenic mechanism in most solid tumors, including hepatocellular carcinoma. Nevertheless, this relationship is not entirely consistent, as a study published in 2020 reported that VEGF expression may occur independently of HIF-1α activation [9]. Emerging evidence suggests that chronic virus-related inflammation contributes to angiogenesis by shaping a pro-tumorigenic microenvironment that facilitates VEGF expression and subsequent tumor progression [10,11].

VEGF expression has been frequently associated with the formation of structurally and functionally abnormal vasculature, which not only supports tumor growth but also fosters an immunosuppressive microenvironment that impairs effective infiltration of effector lymphocytes [12]. Antitumor lymphocyte infiltration, particularly tumor-infiltrating lymphocytes (TILs) and CD4^+^, CD8^+^ T cells, reflects effective immune surveillance and has been consistently associated with improved clinical outcomes in hepatocellular carcinoma in both intratumoral and peritumoral regions [13,14]. Despite growing interest in hypoxia-driven angiogenesis, the association between HIF-1α, VEGF expression and antitumor immune infiltration, especially CD4^+^ and CD8^+^ T-cell dynamics in the post-resection hepatocellular carcinoma microenvironment, has yet to be clearly defined.

Furthermore, hypoxia can interact with immunosuppressive mechanisms by modulating immune checkpoint pathways such as programmed death protein-1 (PD-1) and programmed death ligand-1 (PD-L1). Hypoxia, via HIF-1α, can upregulate PD-L1 expression on tumor cells or regulatory immune cells, thereby contributing to immune tolerance and impairing cytotoxic T-cell activity within the tumor microenvironment [15].

The molecular mechanisms underlying early post-resection recurrence remain incompletely elucidated in the current literature. The interplay between hypoxia via HIF-1α, VEGF, and antitumor immune responses, including TILs, CD4^+^, and CD8^+^ infiltration, as well as pro-tumor mechanisms such as Treg, PD-L1, and PD-1 within both intratumoral and peritumoral regions, and their role in early post-resection recurrence in hepatocellular carcinoma, remains incompletely understood and is poorly characterized in the existing literature. Although several studies have evaluated HIF-1α and VEGF as prognostic biomarkers, findings have been inconsistent. Moreover, emerging evidence indicates that angiogenesis can occur through HIF-1α-independent pathways, highlighting the unresolved and potentially context-dependent role of HIF-1α as a singular mediator of angiogenesis in hepatocellular carcinoma.

## 2. Materials and Methods

### 2.1. Study Design

This multicenter nested case–control study included formalin-fixed paraffin-embedded tumor specimens obtained from 49 patients with HCC who underwent curative surgical resection between 2014 and 2024. Based on surgical criteria, hepatic resection was only indicated for patients with preserved liver function (Child–Pugh class A). Study samples were selected through consecutive sampling. The sample size was determined based on a two-proportion test for RFS at 6 months, with an α of 0.05 and 80% power. For all variables analyzed, the calculated minimum sample size ranged from 24 (for single-proportion analyses) to 48 (for comparisons between two proportions).

Medical records of patients with HCC who underwent primary tumor resection at Cipto Mangunkusumo Hospital, Dharmais Cancer Center Hospital, and Fatmawati Hospital between 2014 and 2024 were retrospectively reviewed. All patients were systematically followed for up to 6 months after curative resection to determine the occurrence of early recurrence. Based on this follow-up, patients were classified into recurrence and non-recurrence groups. All tissue collection and medical records assessments were performed at baseline, immediately after surgical resection and prior to the occurrence of any clinical or radiological evidence of recurrence.

**Figure 1:**
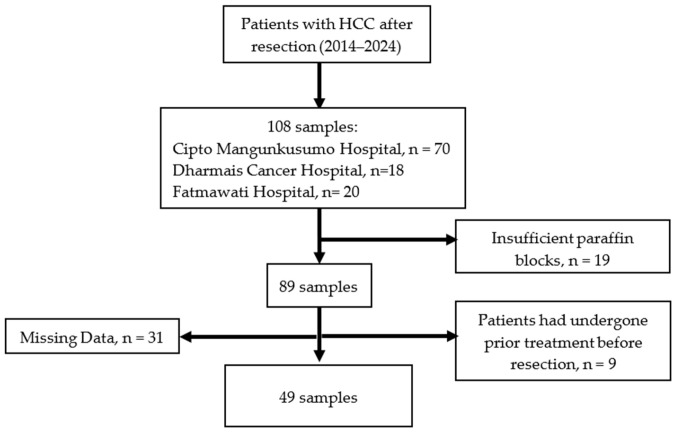
Flow diagram of patient selection.

Of the total 108 patients, 19 samples were excluded due to inadequate tissue for pathological evaluation, 9 were excluded because patients had undergone prior medical interventions before resection, and 31 were excluded due to incomplete clinical data. Consequently, 49 samples that met all eligibility criteria were included in the final analysis. Although the sample size was reduced, strict selection criteria were applied to ensure that only cases with complete data and adequate tissue quality were analyzed. This approach enhanced the consistency and reliability of the study findings, although it limited the number of evaluable cases during the six-month post-resection follow-up period (Figure 1).

### 2.2. Definition of Early Recurrence

Participants were consecutively enrolled and categorized into case (early recurrence ≤6 months) and control (no early recurrence) groups. Early recurrence in this study was operationally defined as recurrence within ≤6 months after curative resection, supported by characteristic findings on triphasic abdominal MRI and serum AFP > 400 ng/mL. Contemporary evidence suggests that shorter time cut-offs provide superior prognostic discrimination between biologically aggressive and indolent recurrence patterns, with 6 months identified as the optimal threshold in post-recurrence survival analyses following curative resection for HCC. This definition was chosen to specifically capture biologically aggressive disease, reflecting early metastatic events or pre-existing micrometastases, which are clinically and biologically distinct from late recurrence occurring beyond this period.

After curative resection, patients were followed according to standard clinical practice. Radiological evaluation using contrast-enhanced CT or MRI was performed approximately 6 months postoperatively to detect possible recurrence. Recurrence data were retrospectively obtained from medical records based on radiological findings.

### 2.3. Tissue Samples

Tissue samples were formalin-fixed and paraffin-embedded. Intratumoral and peritumoral compartments were analyzed, with peritumoral regions defined as non-tumor hepatic stroma within 1000 µm (1 mm) from the tumor margin. The staining procedures were performed according to standardized protocols, and the detailed methodology is provided in Supplementary Table S1.

In this study, the expression of HIF-1α, VEGF, and immune markers was assessed from baseline tumor tissue obtained at the time of surgical resection, and patients were subsequently followed to evaluate recurrence within six months. This approach allows the evaluation of these markers as potential predictors of very early recurrence.

Tumor tissue was obtained at the time of surgical resection, and no repeat tissue sampling was performed during the follow-up period. Routine pathological examination using hematoxylin and eosin (H&E) staining was conducted at the time of surgery. Immunohistochemical (IHC) analysis was not part of routine clinical evaluation; instead, it was performed later during the study using archived formalin-fixed paraffin-embedded tissue blocks. Therefore, all IHC assessments were conducted on specimens collected at a single time point, namely at the time of resection.

### 2.4. Hematoxylin and Eosin (H&E) Staining

TILs were evaluated on H&E-stained sections according to standardized histopathological criteria and categorized based on density within intratumoral and peritumoral compartments.

### 2.5. Immunohistochemistry (IHC)

All paraffin blocks were sectioned at 3 μm thickness using a rotary microtome and mounted on coated glass slides. Slides were incubated at 60 °C for 60 min, followed by deparaffinization in xylene and rehydration through graded alcohols to distilled water. Antigen retrieval was performed using Tris–EDTA buffer (pH 9.0) in a retrieval system (RG-1) for 25 min, followed by cooling at room temperature.

Endogenous peroxidase activity was blocked using hydrogen peroxide or peroxidase blocking solution, and nonspecific binding was prevented using background or protein blocking reagents according to antibody requirements. Slides were incubated with primary antibodies against HIF-1α (rabbit monoclonal EP118, 1:100; Bioscience Technology, Danvers, MA, USA), VEGF (mouse monoclonal clone C-1 sc-7269, 1:100; Santa Cruz Biotechnology, Dallas, TX, USA), PD-1 (mouse monoclonal clone UMAB199, 1:1500; Origene, Rockville, MD, USA), CD8^+^ (rabbit monoclonal clone SP16; Cell Marque, Rocklin, CA, USA), and PD-L1 (mouse monoclonal clone 22C3, 1:50; Dako, Glostrup, Denmark). Primary antibody incubation was performed for 60 min at room temperature or overnight at 4 °C, depending on the antibody.

Signal detection was carried out using polymer-based detection systems (PolyVue Plus or Novolink Polymer) with diaminobenzidine (DAB) as the chromogen. Slides were counterstained with Mayer’s hematoxylin, blued with lithium carbonate, dehydrated, cleared, and mounted with coverslips.

### 2.6. Double Immunohistochemical Staining for FoxP3 and CD4^+^

Double staining was performed using rabbit anti-human FoxP3 (1:50; Epitomics, Burlingame, CA, USA) and mouse anti-human CD4^+^ (clone 4B12; Biocare Medical, Pacheco, CA, USA). Following antigen retrieval and blocking, slides were incubated with both primary antibodies overnight. Detection was conducted using the MACH2 Double Stain system, with DAB and Fast Red as chromogens. Slides were counterstained and mounted using aqueous mounting media.

Clinical variables, HIF-1α and VEGF expression, and immune markers (TILs, CD4^+^, CD8^+^, Treg, PD-1, PD-L1) in intratumoral and peritumoral tissue were obtained at the time of initial surgery, prior to documented recurrence, ensuring temporal validity. Histopathological and immunohistochemical evaluations, including manual cell counts, were performed independently by three pathologists, with no inter-observer discrepancies observed.

### 2.7. Statistical Analysis

Recurrence-free survival (RFS) was defined as the time from the date of curative resection to the first documented recurrence. Survival curves were estimated using the Kaplan–Meier method and compared using the log-rank test. Cut-off values for continuous variables were determined based on previous studies or by maximizing the Youden index (sensitivity + specificity − 1) from receiver operating characteristic (ROC) curves. Data distribution was analyzed using the Mann–Whitney test due to non-normal distribution, with Monte Carlo correction.

Variables with potential association in univariate analysis were subsequently included in a multivariate Cox proportional hazards regression model to identify independent predictors of recurrence. Variables with *p* < 0.25 in univariate analysis were further analyzed using multivariate Cox proportional hazards regression, employing backward likelihood ratio to identify independent predictors of early recurrence. The proportional hazards assumption was satisfied for all variables. No significant collinearity was detected. All statistical analyses were performed using SPSS version 22.0, with 95% confidence intervals. A *p*-value < 0.05 was considered statistically significant.

## 3. Results

### 3.1. Expression of Biomarkers (HE and IHC Results)

#### 3.1.1. TILs Staining

**Figure 2:**
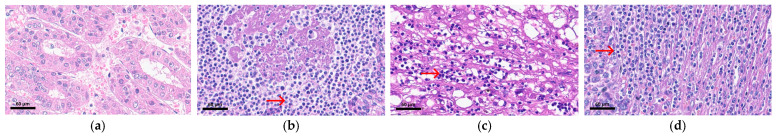
H&E staining of TILs of HCC tissues at 400× magnification. (**a**) Low intratumoral expression in a patient with early recurrence. (**b**) High intratumoral expression in a patient without early recurrence. (**c**) High peritumoral expression in a patient with early recurrence. (**d**) High peritumoral expression in a patient without early recurrence. The red arrows indicate the presence of TILs.

Representative TILs staining is shown in Figure 2.

#### 3.1.2. HIF-1α and VEGF Staining

**Figure 3:**
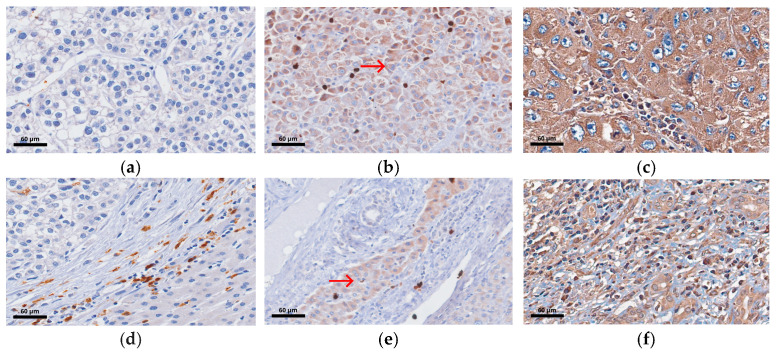
IHC staining of HIF-1α and VEGF expression in intratumoral and peritumoral HCC tissues at 400× magnification. (**a**) Negative intratumoral HIF-1α expression in a patient with early recurrence. (**b**) Positive intratumoral HIF-1α expression in a patient without early recurrence. (**c**) High intratumoral VEGF expression. (**d**) Negative peritumoral HIF-1α expression in a patient with early recurrence. (**e**) Positive peritumoral HIF-1α expression in a patient without early recurrence. (**f**) High VEGF peritumoral expression. Red arrows indicate positive staining of HIF-1α and VEGF.

HIF-1α expression varied among patients, with most recurrent cases showing negative staining, while positive expression was observed in a small proportion of non-recurrent cases. In contrast, VEGF was positive in both intratumoral and peritumoral tissues, with predominantly strong staining intensity. Representative immunohistochemical images are shown in Figure 3a–f.

#### 3.1.3. Immune Cell Infiltration Staining

**Figure 4:**
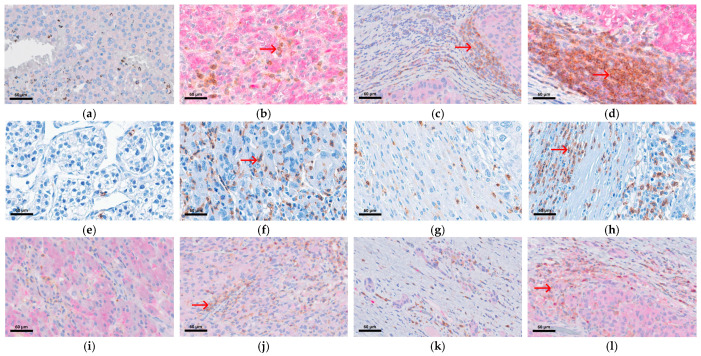
Representative IHC staining of CD4, CD8, and Treg expression in intratumoral and peritumoral areas of HCC tissues at 400× magnification. (**a**) Low intratumoral CD4 expression in patients with early recurrence. (**b**) High intratumoral CD4 staining in in patients without early recurrence. (**c**) High CD4 expression in the peritumoral region of patients with early recurrence. (**d**) High CD4 expression observed in the peritumoral region in patients without early recurrence. (**e**) Low intratumoral CD8 expression in early recurrence patients. (**f**) High intratumoral CD8 expression in patients without early recurrence. (**g**) Low peritumoral CD8 expression in early recurrence HCC. (**h**) High peritumoral CD8 expression in no early recurrence HCC. (**i**) Negative intratumoral Treg expression. (**j**) Positive intratumoral Treg expression. (**k**) Negative peritumoral Treg expression. (**l**) Positive peritumoral Treg expression. Red arrows indicate positive immunostaining of CD4, CD8, and Treg.

Representative intratumoral and peritumoral CD4, CD8, and Treg immunohistochemical staining is shown in Figure 4.

#### 3.1.4. Immune Checkpoint Staining

**Figure 5:**
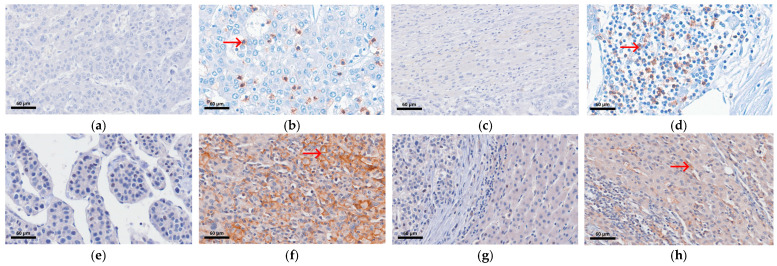
IHC staining of PD-1 and PD-L1 expression in intratumoral and peritumoral regions of HCC tissues at 400× magnification. (**a**) Negative intratumoral PD-1 expression. (**b**) Positive intratumoral PD-1 expression. (**c**) Negative peritumoral PD-1 expression. (**d**) Positive peritumoral PD-1 expression. (**e**) Negative intratumoral PD-L1 expression. (**f**) Positive intratumoral PD-L1 expression. (**g**) Negative peritumoral PD-L1 expression. (**h**) Positive peritumoral PD-L1 expression. Red arrows indicate positive immunostaining of PD-1 and PD-L1.

Representative PD-1 and PD-L1 immunohistochemical staining is shown in Figure 5.

### 3.2. Clinical Characteristics of HCC Patients

A total of 49 patients with hepatocellular carcinoma and Child–Pugh class A liver function were included. Despite surgical resection as first-line therapy, 11 patients (22.4%) experienced very early recurrence within ≤6 months after resection. Patients with early recurrence were generally younger and had larger tumors, although these differences did not reach statistical significance. Microvascular invasion showed a trend toward increased risk, approaching statistical significance. Most patients with early recurrence had underlying liver cirrhosis and viral etiology. No significant differences were observed between groups in AFP, albumin, bilirubin, or platelet levels. Overall, none of the clinical variables were significantly associated with early recurrence, except for microvascular invasion, which demonstrated a near-significant association.

At 1 year post-resection, 15 patients (30.6%) experienced recurrence. No significant differences were observed between case and control groups across all variables (*p* > 0.05). Median AFP was higher in the recurrence group, while other variables showed comparable distributions between groups (Table 1).

### 3.3. ROC Analysis of Immune Markers for Early Recurrence

ROC curve analysis was performed to evaluate the predictive performance of immune markers for early recurrence. The results, including AUC, optimal cut-off values, sensitivity, and specificity, are presented in Table 2.

Intratumoral TILs showed an area under the curve AUC of 0.711 (95% CI: 0.541–0.880) with a *p* value of 0.035 and an optimal cut-off of 65.9, yielding 82% sensitivity and 63% specificity. Peritumoral TILs were not significant (AUC = 0.603, 95% CI: 0.541–0.880; *p* = 0.303), and no optimal cut-off was identified.

Intratumoral CD4^+^ T cells demonstrated an AUC of 0.732 (95% CI: 0.402–0.804; *p* = 0.020) with a cut-off of 27.25, providing 82% sensitivity and 71% specificity. Peritumoral CD4^+^ T cells were not statistically significant (AUC = 0.689, 95% CI: 0.486–0.889; *p* = 0.060).

Intratumoral CD8^+^ T cells showed an AUC of 0.782 (95% CI: 0.630–0.934; *p* = 0.005) with a cut-off of 23.5, sensitivity of 72.7%, and specificity of 81.6%. Peritumoral CD8^+^ T cells were also significant (AUC = 0.703, 95% CI: 0.523–0.884; *p* = 0.042) with a cut-off of 136.4, sensitivity of 72.7%, and specificity of 63.2%.

PD-1 expression in both intratumoral and peritumoral regions showed low predictive performance, with AUCs of 0.272 (95% CI: 0.122–0.421; *p* = 0.002) and 0.243 (95% CI: 0.109–0.377; *p* = 0.001), respectively, and no optimal cut-off values were identified.

### 3.4. Association of Immune Markers with Early Recurrence Within Six Months in Hepatocellular Carcinoma Patients After Resection

The association between immune markers, hypoxia and angiogenesis factors, and early recurrence (≤6 months) was further evaluated using univariate and multivariate analyses. The detailed results are presented in Table 3.

Intratumoral and peritumoral HIF-1α expression were not significantly associated with early recurrence in univariate analysis (*p* = 0.553 and *p* = 0.397, respectively). Similarly, VEGF expression in both compartments showed no significant relationship with early recurrence, although most patients exhibited high VEGF levels.

In univariate analysis, intratumoral CD4^+^ density ≤27.25 was significantly associated with increased risk of early recurrence compared with values >27.25 (unadjusted hazard ratio [UHR] 7.68; 95% CI: 1.66–35.60; *p* = 0.002). Peritumoral CD4^+^ ≤ 20 also demonstrated a significant association with early recurrence (*p* = 0.002). In multivariate Cox regression, intratumoral CD4^+^ remained an independent predictor of early recurrence, with an adjusted hazard ratio (AHR) of 7.98 (95% CI: 1.63–39.07; *p* = 0.001).

Similarly, intratumoral CD8^+^ ≤ 23.5 was associated with a significantly higher risk of early recurrence in univariate analysis (UHR 6.68; 95% CI: 1.77–25.23; *p* = 0.001). Peritumoral CD8^+^ ≤ 136.4 was also significantly associated with early recurrence (UHR 4.20; 95% CI: 1.11–15.91; *p* = 0.021) and remained an independent prognostic factor in multivariate analysis (AHR 4.98; 95% CI: 1.14–21.71; *p* = 0.021).

FoxP3^+^ Tregs, both intratumoral and peritumoral, were not significantly associated with early recurrence in univariate analysis (*p* = 0.609 for both compartments). Intratumoral PD-1 expression did not show a significant relationship with early recurrence (*p* = 0.154). In contrast, peritumoral PD-1 expression was significantly different in univariate analysis (*p* = 0.048), indicating variation in PD-1 distribution in the peritumoral area between patients with and without early recurrence. However, PD-1 peritumoral was not retained in the multivariate Cox model after backward LR elimination. PD-L1 expression, in both intratumoral and peritumoral compartments, was not significantly associated with early recurrence (*p* = 0.298 and *p* = 0.427, respectively) (Table 3).

### 3.5. Recurrence-Free Survival

**Figure 6:**
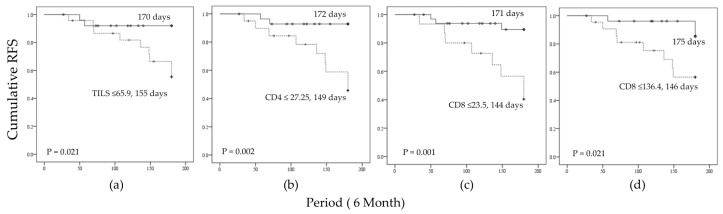
Kaplan–Meier curves of RFS over a 6-month follow-up period after curative resection. Patients were stratified based on (**a**) intratumoral TILs, (**b**) intratumoral CD4 levels, (**c**) intratumoral CD8 levels and (**d**) peritumoral CD8 levels. Mean RFS (in days) for each group is indicated in the figure. Statistical significance between groups was assessed using the log-rank test, with corresponding *p*-values shown in each panel. The dashed line indicates the prognostic cutoff separating patients with poor prognosis (higher risk of early recurrence).

During the six-month follow-up, 11 of 49 patients (22.4%) developed early recurrence, with a mean recurrence-free survival of 162 days. RFS was evaluated over a 6-month period following curative resection (Figure 6).

Lower levels of intratumoral TILs (Figure 6a), intratumoral CD4^+^ (Figure 6b), intratumoral CD8^+^ (Figure 6c), and peritumoral CD8^+^ (Figure 6d) were associated with earlier RFS decline. After adjustment for confounders, only intratumoral CD4^+^ and peritumoral CD8^+^ remained independently associated with early recurrence.

## 4. Discussion

In this study, 11 of 49 (22.4%) of HCC patients experienced early recurrence within six months following curative resection, including those with early-stage disease. These findings underscore that, despite curative resection being the mainstay of therapy, the risk of very early recurrence remains substantial. The proportion of recurrence in this study was slightly lower than that reported in previous studies, where more than 30% of HCC patients experienced recurrence within six months of follow-up [3]. This difference may be attributable to variations in baseline patient characteristics. Prior studies included a higher proportion of patients with poorer prognostic features, such as a greater prevalence of cirrhosis and inclusion of patients with intermediate to advanced BCLC staging system stages [3].

This study demonstrates that patients with early recurrence generally presented with larger tumor size, viral etiology, and moderate-to-poor differentiation. In addition, higher AFP levels were observed in the recurrence group compared with the non-early recurrence group (108 ng/mL [4423.2] vs. 70.0 ng/mL [549.1]). Notably, when stratified by recurrence timing, AFP levels were lower in the ≤6-month group compared with the 1-year group (108 ng/mL [4423.2] vs. 364.1 ng/mL [2673.9]), suggesting that more pronounced AFP elevation is associated with later recurrence rather than very early relapse.

Overall, these findings indicate distinct biological mechanisms underlying different recurrence timeframes. At ≤6 months, MVI emerged as the dominant factor with a strong trend despite borderline significance (*p* = 0.052), supporting the concept that very early recurrence primarily reflects pre-existing micrometastatic disease. Early recurrence was most related to the occult micro-metastasis derived from the initial tumor and correlated with aggressive tumor characteristics, especially microvascular invasion [7,16].

In contrast, at 1 year, the influence of MVI diminished, while the marked increase in AFP may reflect more dynamic tumor biology, tumor evolution, or the possibility of de novo tumorigenesis following curative resection. AFP is a well-established serum biomarker that has long been used in the diagnosis and prognostic evaluation of HCC. Previous studies have demonstrated that AFP is associated with tumor activity, degree of aggressiveness, and invasive and metastatic potential. Elevated AFP levels are often linked to poorer prognosis, including an increased risk of recurrence following curative resection [17].

Our results further demonstrate no significant association between HIF-1α or VEGF expression in either intratumoral or peritumoral tissues and early post-resection recurrence. Notably, all patients who developed early recurrence lacked detectable HIF-1α expression, suggesting that canonical hypoxia-driven transcriptional activation is unlikely to be a dominant driver during the early post-operative period. This finding challenges the prevailing assumption that hypoxia signaling universally underpins aggressive HCC behavior in the immediate post-resection phase. These findings are consistent with previous studies reporting that high HIF-1α expression is rarely observed in HCC, with only 5.56% of patients exhibiting high HIF-1α expression [18].

A study reported that hypoxia has not yet become a dominant factor in patients with early-stage HCC [19,20]. Previous studies have reported that HIF-1α activation increases in parallel with tumor growth and disease progression [21]. Despite the absence of HIF-1α expression, VEGF levels remained high in most early recurrence cases, indicating that angiogenic signaling in HCC may proceed through HIF-1α-independent pathways [22,23]. Biologically, HIF-1α regulates the cellular response to hypoxia and induces multiple target genes involved in angiogenesis, particularly VEGF. Activation of the VEGF pathway promotes neovascularization, contributing to tumor growth, invasion, and metastasis in HCC [24,25,26]. However, clinical evidence indicates that the impact of HIF-1α is not always consistent.

Previous studies have indicated that angiogenesis can occur via HIF-1α-independent mechanisms [9]. Therefore, the high VEGF expression observed in HIF-1α-negative patients in our study likely reflects non-hypoxia-mediated angiogenic pathways, rather than direct activation of HIF-1α. This observation may explain why high VEGF expression was not significantly associated with early post-resection recurrence in our study. Although the majority of patients exhibited elevated VEGF levels, this marker lacked discriminatory power for early recurrence, as many patients without recurrence also demonstrated high VEGF expression. These findings are consistent with previous reports indicating that elevated VEGF expression does not necessarily correlate significantly with recurrence in HCC patients following curative resection [12].

Furthermore, the high VEGF expression observed in this study appears to correspond with the high proportion of patients with viral etiology. Hepatitis B and C virus infections are known to induce chronic hepatic inflammation, which plays a key role in promoting angiogenesis through the upregulation of pro-inflammatory cytokines and growth factors, including VEGF [10].

Increased VEGF expression is known to suppress antitumor immune responses through multiple mechanisms, including inhibition of dendritic cell maturation and impairment of effector T-cell infiltration and function, particularly CD4^+^ and CD8^+^ T cells. In addition, VEGF-induced vascular abnormalities can create a physical barrier that limits the trafficking of TILs into the tumor tissue [27].

Our findings indicate that immune factors may play a more critical biological role than hypoxia. Reduced intratumoral TIL density was associated with earlier recurrence in unadjusted analyses, supporting the role of immune infiltration as a surrogate of effective antitumor immunity. However, this association lost significance after multivariate adjustment, indicating that global TIL counts alone may not sufficiently capture functional immune competence. These findings are consistent with previous reports indicating that low intratumoral TILs serve as a prognostic factor for HCC recurrence [28]. However, after adjusting for potential confounders, the association between intratumoral TILs and early recurrence was no longer statistically significant.

Intratumoral CD4^+^ T cell density was significantly associated with early recurrence in HCC patients during the six-month post-resection follow-up. Patients with high intratumoral CD4^+^ (>27.25) exhibited a median recurrence-free survival of 170 days, whereas the majority of patients who experienced early recurrence had low intratumoral CD4^+^ levels (≤27.25). This association remained significant after adjusting for potential confounders, indicating that intratumoral CD4^+^ T cells serve as an independent predictor of early recurrence.

Intratumoral CD4^+^ T cells play a critical role in both the priming and effector phases of the antitumor immune response, contributing to tumor immunity through direct cytotoxic mechanisms as well as indirect helper-mediated pathways [14]. However, when CD4^+^ T cells and TILs are predominantly localized in the peritumoral compartment and fail to infiltrate the tumor core, local priming and effector activation become inefficient. This limited infiltration reflects the phenomenon of immune exclusion, which impedes direct interactions between immune cells and tumor cells, thereby weakening antitumor immune control and contributing to an increased risk of early recurrence [29].

The observation of reduced intratumoral CD8^+^ T cells in patients with early recurrence, correlating with shorter recurrence-free survival, reinforces the pivotal role of CD8^+^ T cells in controlling residual tumor burden post-surgery. Although this association did not remain significant in the multivariate model for intratumoral CD8^+^, decreased peritumoral CD8^+^ levels retained statistical significance even after adjustment for other variables, highlighting the central role of peritumoral CD8^+^ T cells. The independent association between reduced peritumoral CD8^+^ expression and the risk of early recurrence underscores that peritumoral CD8^+^ depletion contributes independently to early post-resection recurrence, even after accounting for other immunological variables.

Yusa et al. [13] reported that low peritumoral CD8^+^ T cell density serves as a predictor of reduced survival in HCC patients. This finding reflects impaired immune surveillance at the tumor margin, facilitating tumor cell dissemination into surrounding tissues. Similarly, Gabrielson et al. [30] demonstrated that reduced peritumoral CD8^+^ T cells are significantly associated with poorer prognosis in HCC patients. These observations reinforce the interpretation that peritumoral CD8^+^ infiltration constitutes a critical line of defense in suppressing tumor recurrence after resection. Collectively, these findings indicate that diminished cytotoxic T cell infiltration in the peritumoral region compromises the immune system’s ability to control the growth and spread of residual tumor cells post-resection. High levels of CD8^+^ are associated with slower tumor progression and may serve as a prognostic factor in HCC [31].

The uniformly low Treg density observed in both compartments indicates that immunosuppression in early recurrence may not be primarily mediated by regulatory T cell expansion. Instead, immune failure in this setting appears to be driven by insufficient effector immune infiltration rather than active suppression. Consistent with our results, a study concluded that Treg expression was not significantly associated with either recurrence or overall survival in HCC patients [32].

PD-1 and PD-L1 expression did not predict early recurrence. Higher PD-1 expression was predominantly observed in non-recurrent cases, implying that PD-1 positivity may reflect the presence of an active, antigen-experienced immune infiltrate rather than dysfunctional exhaustion in this early-stage setting. The inability of ROC analysis to identify meaningful cut-offs further supports the limited discriminatory value of immune checkpoint markers for very early recurrence. While numerous studies have linked PD-1 and PD-L1 expression to poor prognosis in advanced cancers, their role as predictors of early recurrence after resection remains inconsistent [33]. Furthermore, PD-1 expression was not associated with reduced survival and tumor recurrence in patients with hepatocellular carcinoma [33,34].

These findings are supported by previous studies reporting that increased VEGF drives pathological (disorganized) angiogenesis, characterized by newly formed vessels that are fragile, tortuous, and highly permeable [35]. Despite still providing nutrients and oxygen to residual tumor cells, these abnormal vessels create an endothelial barrier that impairs lymphocyte trafficking. This barrier particularly limits the transmigration of cytotoxic CD8^+^ T cells into the tumor parenchyma [36]. In contrast, CD4^+^ T cells tend to be retained within the peritumoral stroma, partly due to chemokine-mediated retention signals [37]. This pattern is consistent with the immune-excluded tumor microenvironment phenotype, in which CD4^+^ T cells accumulate predominantly in the peritumoral stroma, while CD8^+^ T cells are scarce in both peritumoral and intratumoral compartments [29]. Meanwhile, patients without early recurrence exhibit effective immune infiltration consistent with the immune-inflamed phenotype. This phenotype is characterized by the presence of TILs, CD4^+^, and CD8^+^ cells within the tumor parenchyma, where immune cells closely interact with tumor cells and demonstrate active immune responses, including T-cell activation and pro-inflammatory cytokine production [38].

**Figure 7:**
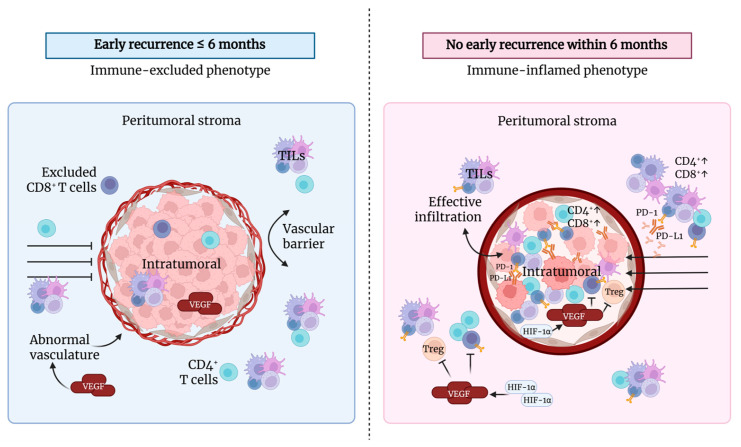
The proposed mechanism illustrates distinct tumor microenvironment phenotypes in HCC. Early recurrence (≤6 months) is characterized by an immune-excluded phenotype, in which CD8^+^ T cells are confined to the peritumoral stroma due to VEGF-mediated abnormal vasculature, with no evidence of activation of HIF-1α signaling. In contrast, patients without early recurrence exhibit an immune-inflamed phenotype, characterized by effective intratumoral infiltration of CD4^+^ and CD8^+^ T cells, active PD-1/PD-L1 interaction, and enhanced antitumor immune responses.

Based on these findings, we propose a pathogenic mechanism in which early recurrence is mediated by non-hypoxia-driven angiogenesis that is HIF-1α-independent, characterized by moderate-to-high VEGF expression despite negative intratumoral and peritumoral HIF-1α. This aberrant angiogenesis results in vascular disorganization and promotes microvascular invasion. The immune landscape exhibits an immune-excluded phenotype, with low intratumoral TILs and CD4^+^ and CD8^+^ cells, while TILs and CD4^+^ cells are trapped in the peritumoral compartment without effective infiltration into the tumor core. The absence of Treg, PD-1 and PD-L1 expression further indicates that immune escape is primarily driven by structural barriers imposed by pathological angiogenesis rather than adaptive immune suppression (Figure 7).

HIF-1α and VEGF alone are inadequate as predictive biomarkers for early recurrence after hepatocellular carcinoma resection. More informative risk stratification should emphasize the immune landscape of the tumor microenvironment with consideration of tissue compartments, as intratumoral CD4^+^ and peritumoral CD8^+^ lymphocytes emerged as independent predictors of very early recurrence (≤6 months) following surgery.

This study has several limitations. First, a complete-case analysis approach was applied; therefore, only 49 patients with complete clinical data were included without imputation of missing values. This approach was chosen to preserve the validity of key variable measurements, although it may have reduced the sample size and statistical power. To minimize overfitting, the multivariate model was restricted to variables with *p* < 0.25 in univariate analysis, in accordance with Hosmer’s recommendations. Median RFS could not be estimated, as the number of early recurrence events had not yet reached half of the study sample.

Second, serum AFP was assessed only at the circulating level without tissue-based evaluation. Therefore, its contribution at the tumor microenvironment level could not be further explored. Furthermore, although this study assessed a broad panel of immune markers, including TILs, CD4^+^, CD8^+^, Treg, PD-1, and PD-L1 in both intratumoral and peritumoral compartments, other immune subsets beyond the scope of this study remain to be investigated in future research.

Third, IHC allows preservation of tissue architecture and spatial context. However, it is limited in terms of quantification and multiplexing capacity. Future studies should therefore integrate multi-omics and spatial profiling approaches, including genomic, transcriptomic, and spatial immune analyses, to better characterize the genetic and immunological alterations associated with early recurrence of HCC. Techniques such as RNA sequencing, single-cell sequencing, and multiplex immunofluorescence may help identify dysregulated molecular pathways, intratumoral heterogeneity, immune cell interactions, and mechanisms of immune evasion that contribute to aggressive tumor behavior and early postoperative recurrence.

Nevertheless, this study provides important evidence highlighting immune depletion and HIF-1α-independent angiogenesis as factors associated with early recurrence of HCC. These findings strengthen the biological basis of early recurrence and provide a foundation for future larger-scale prospective longitudinal studies for validation and generalizability.

## 5. Conclusions

Early recurrence of hepatocellular carcinoma within six months following curative resection reflects a biologically distinct process that is not primarily driven by hypoxia-dependent signaling. The absence of HIF-1α expression in recurrent cases, despite prevalent VEGF upregulation, indicates that early angiogenic activity is largely mediated through HIF-1α-independent molecular pathways and lacks discriminatory value for predicting early post-resection recurrence.

Instead, early recurrence appears to be primarily driven by compartment-specific immune insufficiency within the tumor microenvironment rather than by canonical hypoxia-mediated angiogenic signaling. Significant reductions were observed in intratumoral TILs, intratumoral CD4^+^, and both intratumoral and peritumoral CD8^+^ T cells. After adjustment, reduced intratumoral CD4^+^ T-cell density and decreased peritumoral CD8^+^ T-cell infiltration remained independent predictors of recurrence within six months post-resection, underscoring the complementary roles of immune priming and immune surveillance in controlling residual disease.

Overall, these results highlight that early HCC recurrence is characterized by two key biological features, HIF-1α-independent angiogenesis and spatial immune depletion within the tumor microenvironment. These findings reinforce that molecular risk stratification should not rely solely on canonical hypoxia–angiogenesis pathways but should incorporate integrated immune profiling for a more comprehensive understanding of tumor biology.

## Figures and Tables

**Table 1:** Clinical and demographic characteristics of hepatocellular carcinoma patients.

	6-Month Recurrence			1-Year Recurrence		
	Control (n = 38)	Case (n = 11)	*p*-Value	Control (n = 34)	Case (n = 15)	*p*-Value
Age, years old (IQR)	60 (18.0)	53 (27.0)	0.116 U	61.5 (18)	55 (17)	0.155 U
Gender, n (%)						
Female	12 (31.6)	4 (36.4)	0.773	10 (29.4)	6 (40.0)	0.500
Male	26 (68.4)	7 (63.6)		24 (70.6)	9 (60.0)	
Tumor size, cm, median (IQR)	7 (5.9)	8 (9.0)	0.191 U	7 (6.8)	7.9 (8.1)	0.276 U
Tumor classification, n (%)						
≤5 cm	12 (31.6)	4 (36.4)	0.580	11 (32.4)	5 (33.3)	0.663
>5 cm	26 (69.4)	7 (63.6)		23 (67.6)	10 (66.7)	
Number of tumors, n (%)						
Single	31 (81.6)	11 (100)	0.096	28 (82.4)	14 (93.3)	0.131
Multiple	7 (5.9)	0		6 (17.6)	1 (6.7)	
Microvascular invasion, n (%)						
No	15 (39.5)	1 (9.1)	0.052	12 (35.3)	4 (26.7)	0.340
Yes	23 (60.5)	10 (90.9)		22 (64.7)	11 (73.3)	
Virus etiology, n (%)						0.749
Non viral	12 (31.6)	3 (27.3)	0.837	11 (32.4)	4 (26.7)	
Viral	26 (69.4)	8 (72.7)		23 (67.6)	11 (73.3)	
Liver cirrhosis, n (%)						
No	18 (47.4)	8 (72.7)	0.192	16 (47.1)	10 (66.7)	0.293
Yes	20 (53.6)	3 (27.3)		18 (52.9)	5 (33.3)	
Satellite nodules, n (%)						
No	34 (89.5)	11 (100.0)	0.233	30 (88.2)	15 (100.0)	0.130
Yes	4 (10.5)	0		4 (11.8)	0	
Tumor resection margin, n (%)						
Negative	34 (89.5)	8 (72.7)	0.148	30 (88.2)	12 (80.0)	0.238
Positive	4 (10.5)	3 (27.3)		4 (11.8)	3 (20.0)	
Type 2 diabetes mellitus, n (%)						
No	33 (86.8)	10 (90.9)	0.618	30 (88.2)	13 (86.7)	0.947
Yes	5 (13.2)	1 (9.1)		4 (11.8)	2 (13.3)	
Hypertension, n (%)						
No	23 (60.5)	8 (72.7)	0.621	21 (61.8)	10 (66.7)	0.908
Yes	15 (39.5)	3 (27.3)		13 (38.2)	5 (33.3)	
Fatty Liver, n (%)						
No	37 (97.4)	10 (90.9)	0.441	33 (97.1)	14 (93.3)	0.555
Yes	1 (2.6)	1 (9.1)		1 (2.9)	1 (6.7)	
ECOG performance status, n (%)						
0	15 (39.5)	5 (45.5)	0.541	14 (41.2)	6 (40.0)	0.672
1	23 (60.5)	6 (54.5)		20 (58.8)	9 (60.0)	
BCLC stage, n (%)						
BCLC A	25 (65.8)	6 (54.5)	0.583	23 (67.6)	8 (53.3)	0.512
BCLC B	13 (34.2)	5 (45.5)		11 (32.4)	7 (46.7)	
Tumor differentiation grade, n (%)						
Well differentiated	3 (7.9)	0		2 (5.9)	1 (6.7)	0.984
Moderately differentiated	22 (57.9)	8 (72.7)	0.601	20 (58.8)	10 (66.7)	
Poorly differentiated	13 (39.5)	3 (27.3)		12 (35.3)	4 (26.7)	
AFP, ng/mL, median (IQR)	70.0 (549.1)	108 (4423.2)	0.303 U	56.95 (347.49)	364.1 (2673.9)	0.134 U
Albumin, g/dL, median (IQR)	4.2 (1.0)	3.95 (0.5)	0.484 U	4.19 (1.1)	4 (0.7)	0.403 U
Bilirubin, mg/dL, median (IQR)	0.72 (0.4)	0.60 (0.7)	0.303 U	0.71 (0.4)	0.63 (0.33)	0.573 U
Platelet, 10^3^/µL, median (IQR)	188 (112.8)	280 (190.0)	0.151 U	194.5 (119.0)	184 (159.0)	0.688 U

AFP: Alpha-fetoprotein; BCLC: Barcelona Clinic Liver Cancer; IQR: Interquartile range; U: Mann–Whitney U analysis.

**Table 2:** ROC curve analysis of immune markers for early recurrence.

Parameters	AUC	*p*-Value	95% CI	Cut off	Sensitivity (%)	Specificity (%)
TILs Intratumoral	0.711	0.035	0.541–0.880	65.9	82.0	63.0
TILs Peritumoral	0.603	0.303	0.402–0.804	262.10	81.8	42.1
CD4^+^ Intratumoral	0.732	0.020	0.570–0.894	27.25	82.0	71.0
CD4^+^ Peritumoral	0.689	0.060	0.486–0.889	117.88	72.7	60.5
CD8^+^ Intratumoral	0.782	0.005	0.630–0.934	23.5	72.7	81.6
CD8^+^ Peritumoral	0.703	0.042	0.523–0.884	136.4	72.7	63.2
PD-1 Intratumoral	0.272	0.002	0.122–0.421	NA	NA	NA
PD-1 Peritumoral	0.243	0.001	0.109–0.377	NA	NA	NA

AUC: Area Under the Curve; CI: Confidence Interval; NA: Not Available.

**Table 3:** Association of TILs, HIF-1α, VEGF, CD4^+^, CD8^+^, PD-1, and PD-L1 with early recurrence within six months post-resection in hepatocellular carcinoma patients.

Parameters		Early Recurrence		Univariate Hazard Ratio (95% CI)	UPV	Multivariat Hazard Ratio (95% CI)	MPV
		Control (n = 38)	Case (n = 11)				
TILs	Intratumoral, n (%)						
	>65.9	24 (63.2)	2 (18.2)	Reference (1.00)	0.021		
	≤65.9	14 (36.8)	9 (81.8)	5.02 (1.09–23.26)			
	Peritumoral, n (%)						
	>40	38 (100)	9 (81.8)		0.007		
	≤40	0	2 (18.2)				
HIF 1-α	Intratumoral, n (%)						
	Negative	32 (84.2)	11 (100)		0.553		
	Moderate	3 (7.9)	0				
	High	3 (7.9)	0				
	Peritumoral, n (%)						
	Negative	31 (81.6)	11 (100)				
	Moderate	2 (5.3)	0		0.397		
	High	5 (13.2)	0				
VEGF	Intratumoral, n (%)						
	Weak	3 (7.9)	1 (9.1)				
	Moderate	4 (10.5)	3 (27.3)		0.123		
	High	31 (81.6)	7 (63.6)				
	Peritumoral, n (%)						
	Weak	2 (5.3)	1 (9.1)				
	Moderate	4 (10.5)	3 (27.3)		0.059		
	High	32 (84.2)	7 (63.6)				
CD4	Intratumoral, n (%)						
	>27.25	27 (71.1)	2 (18.2)	Reference (1.00)	0.002	Reference (1.00)	
	≤27.25	11 (28.9)	9 (81.8)	7.68 (1.66–35.60)		7.98 (1.63–39.07)	0.001
	Peritumoral, n (%)						
	>20	38 (100)	9 (81.8)		0.002		
	≤20	0	2 (18.2)				
CD8	Intratumoral, n (%)						
	>23.5	31 (81.6)	3 (27.3)	Reference (1.00)	0.001		
	≤23.5	7 (18.4)	8 (72.7)	6.68 (1.77–25.23)			
	Peritumoral, n (%)						
	>136.4	24 (63.2)	3 (27.3)	Reference (1.00)	0.021	Reference (1.00)	
	≤136.4	14 (36.8)	8 (72.7)	4.20 (1.11–15.91)		4.98 (1.14–21.71)	0.021
Treg	Intratumoral, n (%)						
	Negative	35 (92.1)	10 (90.1)		0.609		
	Positive	3 (7.9)	1 (9.9)				
	Peritumoral, n (%)						
	Negative	36 (94.7)	10 (90.1)		0.609		
	Positive	2 (5.3)	1 (9.9)				
PD-1	Intratumoral, n (%)						
	Negative	15 (39.5)	9 (81.8)		0.154		
	Low	8 (21.1)	1 (9.1)				
	Moderate	7 (18.4)	1 (9.1)				
	High	8 (21.1)	0				
	Peritumoral, n (%)						
	Negative	15 (39.5)	9 (81.8)		0.048		
	Low	3 (7.9)	2 (18.2)				
	Moderate	12 (31.6)	0				
	High	8 (21.1)	0				
PD-L1	Intratumoral, n (%)						
	Negative	29 (76.3)	11 (100)				
	Moderate	7 (18.4)	0		0.298		
	High	2 (5.3)	0				
	Peritumoral, n (%)						
	Negative	35 (92.1)	11 (100)		0.427		
	Moderate	3 (7.9)	0				

MPV: Multivariate *p* value; UPV: Univariate *p* value.

## Data Availability

The data presented in this study are not publicly available due to patient privacy and ethical restrictions but are available from the corresponding author upon reasonable request.

## Supplementary Materials

The following supporting information can be downloaded at: https://www.mdpi.com/article/10.3390/biom16050723/s1. Supplementary Table S1. Methodological procedures and scoring criteria for hematoxylin–eosin (H&E) and immunohistochemistry (IHC) staining of TILs, HIF-1α, VEGF, CD4, CD8, Treg, PD-1, and PD-L1.

## Abbreviations

The following abbreviations are used in this manuscript:

AFP	Alpha-fetoprotein
BCLC	Barcelona Clinic Liver Cancer
CD	Cluster of Differentiation
ECOG	Eastern Cooperative Oncology Group
FOXP3	Forkhead box P3
HCC	Hepatocellular Carcinoma
HIF	Hypoxia-inducible factor
MRI	Magnetic resonance imaging
PD1	Programmed Cell Death Protein 1
PDL1	Programmed death-ligand 1
TILs	Tumor-infiltrating lymphocytes
VEGF	Vascular Endothelial Growth Factor
