# Supplementary material for: Early Recurrence of HCC Is Driven by Inflammation-Related HIF-1α Independent Angiogenesis Rather than Hypoxia-Induced Immune Escape
Source: Biomolecules. 2026 May 14;16(5):723. doi: 10.3390/biom16050723 (PMC13204095; doi:10.3390/biom16050723)
Supplement: Supplementary file 1 [file biomolecules-16-00723-s001.zip › biomolecules-4172385-supplementary.pdf]

Supplementary

Table S1. H&E and Immunohistochemistry Variables

Variables	Type of methods	Methodological Procedures
Tumor infiltrating lymphocytes (TIL)	H&E	• For diagnostic purposes, only TILs found in stromal area were considred relevant.<br>• The assessment of TILs was performed as the percentage of the stromal area, excluding regions containing carcinoma cells from the total evaluated surface area. Images were acquired at 200-400 magnification. Divided into three groups based on the percentage of TILs within the stromal area, namely:<br>▪ Group A (No/Minimal): 0-10%<br>▪ Group B (Intermediate/Heterogen): 10-40%<br>▪ Group C (High): 40-90%<br>• Lymphocytic infiltration was also assessed in 10 high-power fields (HPFs) at 400× magnification, and the average lymphocyte infiltration was calculated, with ≤50 TILs/HPF considered as low expression and >50 TILs/HPF as high expression.
CD4/CD8	IHC	• A single 4-μm paraffin block<br>• IHC was performed using CD4 and CD8 antibodies (mouse anti-CD4 and anti-CD8 monoclonal antibodies).<br>• The density of $CD4^+$ and $CD8^+$ T lymphocytes was evaluated in 10 high-power fields (HPFs) at 200 magnification for both intratumoral and peritumoral regions.<br>• The five areas with the highest number of immune cells were then examined at 400× magnification to quantify immunoreactive cells, and the counts were averaged across these five fields for classification as follows:<br>• CD4 >20 were defined as CD4(+)<br>• CD8 >100 were defined as CD8 (+)
VEGF	IHC	• IHC was performed using Anti-VEGF mouse monoclonal antibody, clone C-1 sc-7269 (Santa Cruz biotechnology)<br>• The percentage of VEGF-positive cells was assessed in 10 high-power fields (HPFs) at 200-400 magnification.<br>• IHC scoring was performed semi-quantitatively with dual assessment. VEGF expression levels were evaluated based on staining intensity according to the following criteria:<br>▪ 0 = No staining<br>▪ 1 = Weak staining<br>▪ 2= Moderate staining<br>▪ 3 = Strong staining
HIF-1α	IHC	• IHC was performed using Anti-HIF-1a antibody (clone H1alpha67, Santa Cruz Biotechnology, CA, USA)

		• Assessment was performed in 10 high-power fields (HPFs) at 400× magnification, with 1,000 cells counted from each core.<br>• Protein levels were categorized based on the percentage of cells exhibiting cytoplasmic and/or nuclear staining using the following classification system:<br>▪ Negative : No staining<br>▪ Low: ≤10% of cells positive for nuclear and/or weak cytoplasmic staining<br>▪ Moderate: 10–50% of cells positive for nuclear and/or distinct cytoplasmic staining<br>▪ High : >50% of cell positives for nuclear and/or strong cytoplasmic staining
CD4-FoxP3	IHC	• IHC was performed using Immune related antibody (FoxP3 259D)<br>• Three of the most representative and independent fields were selected at 200-400 magnification. Using the FoxP3/CD4 ratio:<br>▪ Positive = > 5%<br>▪ Negative = ≤ 5%
PD-1	IHC	• Analysis was conducted to determine PD-1 expression in tumor tissue.<br>• *Mouse monoclonal anti-human antibody* PD-1<br>• 3-Amino-9-ethylcarbazole (red) was used as the substrate, followed by hematoxylin as a counterstain.<br>• Three independent pathologists examined the paraffin slides blinded to clinical information to evaluate PD-1 staining.<br>• PD-1 staining intensity was evaluated and classified into four levels based on the proportion of positive tumor cells.<br>▪ Negative: <1% PD-1–positive tumor cells<br>▪ Low: 1–24% PD-1–positive tumor cells<br>▪ Moderate: 25–49% PD-1–positive tumor cells<br>▪ High: ≥50% PD-1–positive tumor cells
PDL-1	IHC	• Analysis was performed to assess PD-L1 expression in tumor tissue.<br>• *Mouse monoclonal anti-human antibody* PD-L1<br>• 3-Amino-9-ethylcarbazole (red) was used as the substrate, followed by hematoxylin as a counterstain.<br>• Three independent pathologists examined the paraffin slides blinded to clinical information to evaluate PD-L1 staining.<br>▪ PD-L1 staining intensity was evaluated and classified into four levels based on the intensity of the positive staining area.<br>▪ Negative : <1% PD-L1–positive tumor cells<br>▪ Moderate : 1-49% PD-L1–positive tumor cells<br>▪ High : ≥50% PD-L1–positive tumor cells
